# Supplementary material for: Implementation Models of Compassionate Communities and Compassionate Cities at the End of Life: A Systematic Review
Source: Int J Environ Res Public Health. 2020 Aug 28;17(17):6271. doi: 10.3390/ijerph17176271 (PMC7504622; doi:10.3390/ijerph17176271)
Supplement: Supplementary file 1 [file ijerph-17-06271-s001.pdf]

Table S1: Characteristics of included studies.

Author, year	Aims	Study design	Participants/scope	Information contained	Quality assessment (GRADE)	Country	Main findings
Kayser et al., 2005 [23]	To investigate the factors that influence the quality of care of residents with needs of end-of-life care admitted to the hospice.	Descriptive study.	33 patients with end-of-life care needs.	Models of Compassionate Communities and Compassionate Cities at the end of life.	Low	United Kingdom	Care of people at the end of life must be provided by 3 components: Care, Compassion and Community.
Chou et al., 2008 [20]	To describe the program Chinese-American Coalition for Compassionate Care	Descriptive study.	Not apply	Models of Compassionate Communities and Compassionate Cities at the end of life.	Very Low	USA	This article describes the Chinese American Coalition for Compassionate Care as a collective model of community.
Blinderman, 2009 [22]	To describe the need of palliative care as a public health priority in countries with low resources.	Descriptive study.	Population with cancer, HIV, advanced chronic diseases, aging of the population, etc. Need for CP in public health programs.	Models of Compassionate Communities and Compassionate Cities at the end of life.	Very Low	Poor countries	Analysis, from the bioethical principles, about the importance of treating palliative care as a priority in public health. It does not propose strategies, assessment sysems or ways to carry it out.
Flager & Dong, 2010 [25]	To develop a critical analysis of the access and coverage of the program.	Descriptive study.	Compassionate Care Benefit (CCB) Canada model.	Models of Compassionate Communities and Compassionate Cities at the end of life.	Low	Canada	National regulations and public health models establish strategic lines for the development of communities and compassionate cities. A compassionate program that supports the entire population of caregivers equally, regardless of their work situation it´s necessary.

Author, year	Aims	Study design	Participants/scope	Information contained	Quality assessment (GRADE)	Country	Main findings
Sallnow et al., 2010 [21]	To describe the operation and scope of a community program.	Descriptive study.	Not apply.	Models of Compassionate Communities and Compassionate Cities at the end of life.	Very low	India	Acting beyond the traditional medical model allows greater coverage, a more comprehensive approach to the patient and generates social commitment.
Abel J, 2011 [17]	To explore how local social resources can be mobilized based on compassionate communities.	Descriptive study.	Weston-super-Mare.	Models of Compassionate Communities and Compassionate Cities at the end of life.	Very Low	United Kingdom.	This model motivates social changes of behaviors and attitudes towards death, and guarantees basic care to people at the end of life.
Abel et al., 2013 [24]	To describe a model of care which set patients at the centre of a network which includes inner and outner networks, communities and service delivery organisations.	Descriptive study.	Not apply.	Models of Compassionate Communities and Compassionate Cities at the end of life.	Low	United Kingdom.	Communities can be supporters of care for people at the end of their lives enhancing meaning and value for both patients and community member envolved.
Kellehear, 2013 [26]	To examine the new policy and practice development in British end-of-life care explaining its conceptual origins and describing its policy importance to current practice.	Descriptive study.	Hospices from England and Japon.	Models of Compassionate Communities and Compassionate Cities at the end of life.	Very Low	United Kingdom.	Care of people at the end of life is everyone's responsibility. Each person in each community has a role; as well as the governmental, educational and social organizations. Compassionate cities and communities are an example of health responsibility.

**Author, year**	**Aims**	**Study design**	**Participants/scope**	**Information contained**	**Quality assessment (GRADE)**	**Country**	**Main findings**
Paul & Sallnow, 2013 [18]	To determine if such public health approaches were a priority for UK palliative care services, and to obtain a baseline of current initiatives.	Descriptive study.	4 UK countries. 220 palliative care providers.	Models of Compassionate Communities and Compassionate Cities at the end of life.	Low	United Kingdom.	60% of services identified public health approaches to end-of-life care as a priority for their service. Working with schools was the most common project undertaken by services identified.
Lown, 2014 [16]	To present a set of guiding commitments and recommendations to foster a more compassionate healthcare system.<br>To urge healthcare organizations to adopt these commitments and take action to embed compassionate care in all aspects of training, research, patient care and organizational life.	Descriptive study.	Not apply.	Models of Compassionate Communities and Compassionate Cities at the end of life.	Low	USA	The guide commitments ask all healthcare organizations to take action to embed compassionate care in all aspects of training, research and organizational aspects.
De Zulueta, 2016 [19]	To integrate the perspectives of science and psychology with modern leadership and health organizations theories, in an effort to arrive at a useful synthesis "".	Integrative review.	Not apply.	Models of Compassionate Communities and Compassionate Cities at the end of life.	Very Low	United Kingdom	This article exposes the need of compassionate models and the need of paradigms changing to centralize medicine in care (with adequate training in the faculties) and comumitary education.<br>It does neither specify strategies, nor raises models, application or evaluation.

Author, year	Aims	Study design	Participants/scope	Information contained	Quality assessment (GRADE)	Country	Main findings
Luzinski et al., 2008 [29]	To evaluate a program of Community Case Coordinators.	Intervention study.	400 patients/ year	Evaluation models of Compassionate Communities and Compassionate Cities: Indicators, standars, or data that allow evaluating process and results.	Low	USA	This program generates economic benefits and quality of service for those who implement them. Interventions by the case managers saved an average of $ 93,000 per year.
Giesbrecht et al., 2010 [34]	To gather input from key stakeholders in order to develop policy-relevant recommendations that can help the programme to better meet the needs of Canadian family caregivers.	Qualitative study.	50 palliative care providers.	Evaluation models of Compassionate Communities and Compassionate Cities: Indicators, standars, or data that allow evaluating process and results.	Low	Canada	Findings from thematic analysis reveal four specific sub-themes of expectation: (1) temporal; (2) financial; (3) informational, and (4) administrative.
Williams et al., 2011 [31]	To examine how Canada's (CCB) operates as a public health response in sustaining informal caregivers providing P/EOL care, and whether or not it adequately addresses known aspects of caregiver burden that are addressed within the population health promotion model.	Qualitative study.	57 caregivers of people at the end of life.	Evaluation models of Compassionate Communities and Compassionate Cities: Indicators, standars, or data that allow evaluating process and results.	Low	Canada	CCB has great potential to contribute to aspects that cause claudication or fatigue in the caregivers; this program should consider improving strategies that help to reconcile work activity with patient care, as well as financial and emotional support.

Author, year	Aims	Study design	Participants/scope	Information contained	Quality assessment (GRADE)	Country	Main findings
Williams, 2010 [32]	To evaluate the Compassionate Care Model.	Qualitative study.	25 telephone interviews of the benefits caregivers of CC	Evaluation models of Compassionate Communities and Compassionate Cities: Indicators, standars, or data that allow evaluating process and results.	Low	Canada	The article describes a pilot study carried out to evaluate an analysis tool, in order to use it in the evaluation study of the CCB. The article exposes a useful tool to evaluate models and programs such as the CCB.
Crooks et al., 2012 [30]	To know the expectations of the program with the purpose of exposing relevant recommendations to be taken into account by the entities involved in the development of the program.	Qualitative study.	57 caregivers of people at the end of life.	Evaluation models of Compassionate Communities and Compassionate Cities: Indicators, standars, or data that allow evaluating process and results.	Low	Canada.	This analysis plays an important role in identifying potential changes for the Compassionate Communities Benefits. The program provides better support for family caregivers.
Giesbrecht et al., 2012 [33]	To examine family caregiving at the end-of-life from the perspective of formal front-line palliative care providers (e.g., community nurses, social workers) in order to gain a better understanding of the axes of difference directly impacting family caregivers' support opportunities, access, and outcomes.	Qualitative study.	50 palliative care providers.	Evaluation models of Compassionate Communities and Compassionate Cities: Indicators, standars, or data that allow evaluating process and results.	Low	Canada.	Findings helps redefine the application of the program (CCB) to achieve better coverage; It states that there are several caregivers who require more care and attention because they are in more vulnerable conditions, and a vulnerable caregiver conditions vulnerability in the one who receives the care.

Author, year	Aims	Study design	Participants/scope	Information contained	Quality assessment (GRADE)	Country	Main findings
Shih et al., 2012 [40]	To assess the impact of a 1-day compassion-focused palliative care training course on preclinical medical students' knowledge of clinical management and perception of compassionate care, and their beliefs regarding ethical decisions. To identify what factors were most influential in improving the ease of the decision-making process.	Intervention study.	251 preclinical medical students were enrolled in a palliative care training course.	Evaluation models of Compassionate Communities and Compassionate Cities: Indicators, standars, or data that allow evaluating process and results.	Low	Taiwan.	A medical compassion training program would allow professionals to improve their skills in order to make the most appropriate decisions from the ethical point of view in patients at the end of life.
Crowther et al., 2013 [36]	To explore the experiences of people with dementia in the last year of life and time surrounding death and how the presence and lack of compassion, kindness and humanity influenced the experience of care.	Qualitative study.	40 bereaved carers of dementia carers.	Evaluation models of Compassionate Communities and Compassionate Cities: Indicators, standars, or data that allow the evaluation process and results.	Low	United Kingdom.	Authors point the necessity of compassion, humanity and kindness in the care of patients to provide adecuate interventions at the end of life. Authors defend that compassionate and human care should be trained in health personnel and general society.

Author, year	Aims	Study design	Participants/scope	Information contained	Quality assessment (GRADE)	Country	Main findings
Martins et al., 2013 [37]	To describe the development of a brief and simple scale for the objective assessment of compassion with specific domains that can be taught and tested across social networks and relationships.	Descriptive study.	Tested in 310 professionals.	Evaluation models of Compassionate Communities and Compassionate Cities: Indicators, standars, or data that allow evaluating process and results.	Low	USA	This scale consists of 10 items to measure compassion. This scale can be used to promote understanding of the impact of the level of compassion in the disease, important aspect in interpersonal relations and community.
McLoughlin, 2013 [35]	"To enrich and support society to live compassionately with death, dying, loss and care and to demonstrate and evaluate the process of developing a compassionate community model in an Irish context."	Descriptive study.	Not apply.	Evaluation models of Compassionate Communities and Compassionate Cities: Indicators, standars, or data that allow evaluating process and results.	Low	Ireland	Indicators of structure and processes have been designed to evaluate a specific compassionate model.
Pesut et al., 2017 [9]	To improve quality of life by developing independence, engagement, and community connections in palliative clients and families in Western context through a three days workshop.	Intervention study.	7 volunteers were partnered with 18 clients.	Evaluation models of Compassionate Communities and Compassionate Cities: Indicators, standars, or data that allow evaluating process and results.	Low	Canada	Clients and family rated the service as highly important to their care. The program was perceived to be well designed by stakeholders and an important need in the community. Sustainability, however, was a concern expressed by both clients and volunteers.

Author, year	Aims	Study design	Participants/scope	Information contained	Quality assessment (GRADE)	Country	Main findings
Sallnow et al., 2016 [27]	To determine the impact of a health-promoting or new public health approach to end-of-life care, specifically as this applies to efforts to strengthen community action.	Systematic review.	8 studies relating to community action in end-of-life care.	Evaluation models of Compassionate Communities and Compassionate Cities: Indicators, standars, or data that allow evaluating process and results.	Low	Ireland	Communities' action in end of life have beneficially impacts on carers and family members, people with a life-limiting illness and individuals in the wider community.
Sinclair et al., 2016 [38]	To map out the field of study on compassion in healthcare and identify gaps in the existing evidence base. To provide recommendations that will inform future research in the areas of theory, education, research, and clinical practice.	Scoping review.	44 Studies were included.	Evaluation models of Compassionate Communities and Compassionate Cities: Indicators, standars, or data that allow evaluating process and results.	Low	Canada	Six themes emerged from studies that explored perceptions of compassionate care: nature of compassion, development of compassion, interpersonal factors related to compassion, action and practical compassion, barriers and enablers of compassion, and outcomes of compassion. Clinicians and students described many healthcare system barriers that diminished their potential for compassion, including a lack of time, support, staffing, and resources.Compassion was also associated with positive clinician outcomes, including increased job satisfaction and sustainment.

Author, year	Aims	Study design	Participants/scope	Information contained	Quality assessment (GRADE)	Country	Main findings
Sinclair et al., 2016 [39]	To investigate palliative cancer patients' understanding and experiences of compassion to provide a critical perspective on the nature and importance of compassion.	Descriptive study.	53 Adult cancer inpatients on a palliative care unit and a hospital palliative care consult service at a large acute academic hospital.	Evaluation models of Compassionate Communities and Compassionate Cities: Indicators, standars, or data that allow evaluating process and results.	Low	Canada.	The key elements of compassion emerging from the data generated seven categories, Virtues, Relational space, Virtuous response, Seeking to Understand, Relational Communicating, Attending to needs, Patients reported outcomes.
Pfaff & Markaki, 2017 [28]	To identify Compassionate Collaborative Care quality indicators through a systematic review.	Systematic review.	Not apply.	Evaluation models of Compassionate Communities and Compassionate Cities: Indicators, standars, or data that allow evaluating process and results.	Low	USA	The great contribution of this review is a synthesis of the importance of the Compassionate Collaborative Care: improve health, strengthen the provision of care and controls health costs.
Dewar & Cook, 2014 [41]	To support staff to work together to develop a culture of inquiry that would enhance delivery of compassionate care.	Descriptive study.	86 nursing staff covering 24 in-patient areas within one acute NHS Board in Scotland.	Tools, protocols or information systems for evaluation of Compassionate Communities and Compassionate Cities.	Low	Scotland.	78% of participants reported enhanced self-awareness. 93% talked about enhanced relationships. 58% reported more opportunities to reflect on their practice. 98% reported using different conversations in the workplace. 69% made comments about being motivated to learn about others' experiences and use this as a platform for continuous improvement

Author, year	Aims	Study design	Participants/scope	Information contained	Quality assessment (GRADE)	Country	Main findings
Elliot et al., 2014 [43]	-To understand how the Compassion Intervention operates in practice, in two care homes, in two different health and social care economies.<br>-To collect process and outcome data on feasibility, acceptability and economic costs in order to inform the planning of a future phase II trial.	Descriptive study.	People with dementia in care home settings.	Tools, protocols or information systems for evaluation of Compassionate Communities and Compassionate Cities.	Low	United Kingdom.	Article describes three overarching core components of the Compassion Intervention: Facilitation of integrated care; Education, training and support; Investment from commissioners and care providers.
McLoughlin et al., 2015 [44]	-To develop a greater understanding of the practical and social needs of people living with advanced life limiting illness.<br>-To assess the feasibility, acceptability and subsequent effectiveness of The Good Neighbour Partnership, a volunteer-led model of social and practical care/support for community dwelling adults living with advanced life limiting illness.	Descriptive study.	Protocol for adults living with a life-limiting illness considered by a member of the primary care/hospice at home team to be in their last year of life and/or their carer.	Tools, protocols or information systems for evaluation of Compassionate Communities and Compassionate Cities.	Low	Ireland.	INSPIRE protocol is an effectiveness, efficacy, utility and acceptability model of social and practical care for people with life-limiting illness.

Author, year	Aims	Study design	Participants/scope	Information contained	Quality assessment (GRADE)	Country	Main findings
Walshe et al., 2016 [45]	To determine if receiving care from a social action volunteer befriending service plus usual care significantly improves quality of life in the last year of life.	Descriptive study.	12 individual sites across England will participate in this study.	Tools, protocols or information systems for evaluation of Compassionate Communities and Compassionate Cities.	Low	United Kingdom.	A protocol that uses models of social action can serve to support end-of-life care in community settings and allows the evaluation of the experience and opinions of patients and family members. The use of this protocol can help reduce isolation, meet the emotional needs of patients and make sense of community involvement.
Moore et al., 2017 [42]	To understand how a "Compassion intervention" operated in two nursing homes and estimate the cost of employing an Interdisciplinary Care Leader to facilitate the intervention.	Intervention study.	Two nursing homes. 30 residents with advanced dementia were assessed of whom 9 were recruited for data collection; 4 of these residents' family members were interviewed. 28 nursing home and external healthcare professionals participated in interviews at 7 (n=19), 11 (n=19) and 15 months (n=10).	Tools, protocols or information systems for evaluation of Compassionate Communities and Compassionate Cities.	Low	United Kingdom.	The Intervention prompted improvements in advance care planning, pain management and person-centred care. Six-month Interdisciplinary care leader costs were £18255.
